# Supplementary material for: Splice-Junction-Based Mapping of Alternative Isoforms in the Human Proteome
Source: Cell Rep. Author manuscript; Available in PMC 2020 Jan 15. (PMC6961840; doi:10.1016/j.celrep.2019.11.026)
Supplement: 2 [file NIHMS1546469-supplement-2.zip › DF1/PXD010154/Prostate/Prostate_13_RETREG2_GLPTGAGGGPGMGLSLGLGLGLSLGMSEATSEAE.pdf]

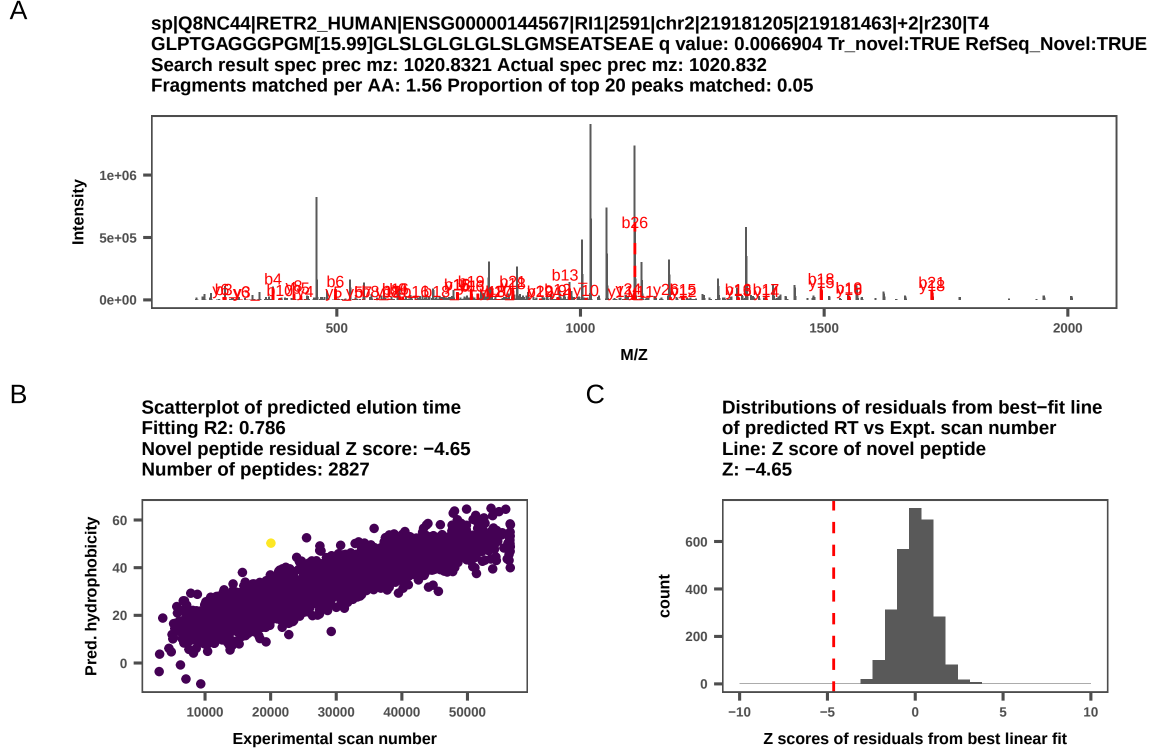

A
sp|Q8NC44|RETR2_HUMAN|ENSG00000144567|RI1|2591|chr2|219181205|219181463|+2|r230|T4
GLPTGAGGGPGM[15.99]GLSLGLGLGLSLGMSEATSEAE q value: 0.0066904 Tr_novel:TRUE RefSeq_Novel:TRUE
Search result spec prec mz: 1020.8321 Actual spec prec mz: 1020.832
Fragments matched per AA: 1.56 Proportion of top 20 peaks matched: 0.05
Intensity
1e+06
5e+05
0e+00
b26
b13
b4
b6
500
1000
1500
2000
M/Z
B
Scatterplot of predicted elution time
Fitting R2: 0.786
Novel peptide residual Z score: −4.65
Number of peptides: 2827
Pred. hydrophobicity
60
40
20
0
10000
20000
30000
40000
50000
Experimental scan number
C
Distributions of residuals from best−fit line
of predicted RT vs Expt. scan number
Line: Z score of novel peptide
Z: −4.65
count
600
400
200
0
−10
−5
0
5
10
Z scores of residuals from best linear fit
